# Dame Cicely Saunders: Pioneering Palliative Care and the Evolution of Hospice Services

**DOI:** 10.7759/cureus.75176

**Published:** 2024-12-05

**Authors:** Hannah M Redwine, Latha Ganti

**Affiliations:** 1 Research, Orlando College of Osteopathic Medicine, Winter Garden, USA; 2 Medical Science, The Warren Alpert Medical School of Brown University, Providence, USA

**Keywords:** historical vignette, hospice, legacy, patient autonomy, quality of life

## Abstract

Dame Cicely Saunders was a pioneer in palliative care and the founder of the modern hospice movement. Her visionary work, particularly in establishing St. Christopher’s Hospice in 1967, reshaped the way healthcare professionals approach the care of patients with life-limiting illnesses. She emphasized a holistic approach that addresses not only physical pain but also the emotional, social, and spiritual needs of patients. Her contributions have left an indelible mark on the medical field, leading to the global expansion of palliative care services and their integration into various medical specialties. Today, palliative care and hospice services are recognized for enhancing the quality of life for patients and their families, focusing on quality over quantity of life, promoting dignity in dying, respecting patient autonomy, and supporting patients in creating a meaningful legacy.

## Introduction and background

Early life and education

Cicely Saunders (1918-2005) was born on June 22, 1918 into a middle-class family in Barnet, London. She received her early education at Roedean School and initially studied politics, philosophy, and economics at St Anne’s College, Oxford. However, her passion for caring for others led her to train as a nurse at St. Thomas' Hospital during World War II [1]. Saunders’ encounters with terminally-ill patients during her nursing career profoundly impacted her, highlighting the deficiencies in care for the dying and igniting her resolve to find better ways to manage pain and provide comfort to those facing the end of their lives [2].

Founding St. Christopher’s Hospice

In the 1950s, Saunders expanded her knowledge by studying social work, and later pursued and earned her medical degree in 1957. This diverse background gave her a unique perspective on patient care. In 1967, she founded St. Christopher’s Hospice in London, the first modern hospice to integrate clinical care with teaching and research [3]. St. Christopher’s quickly became a model for palliative care worldwide, demonstrating that effective pain management combined with compassionate care, emotional and spiritual support could greatly improve the quality of life for patients nearing death [4].

## Review

Palliative care principles and their evolution

Saunders introduced the concept of “total pain,” a term she used to describe the complex nature of suffering experienced by terminally-ill patients. Total pain includes physical discomfort as well as psychological, social, and spiritual distress. This holistic approach became the cornerstone of palliative care, which prioritizes improving the quality of life for patients and their families over merely extending their lives [5].

The principles Saunders established have evolved over time, leading to the adoption of palliative care services worldwide. Today, there are over 8,000 palliative care programs in hospitals, nursing homes, and hospice organizations globally, with significant growth seen in low- and middle-income countries [6].

Multidisciplinary approach in palliative care

One of Saunders’ most significant contributions to medicine is the establishment of the multidisciplinary approach in palliative care. This approach acknowledges that addressing the complex needs of patients with life-limiting illnesses requires a team of professionals with varied expertise. A multidisciplinary team typically includes physicians, nurses, social workers, chaplains, psychologists, and volunteers, each contributing their skills to support both the patient and their family [7].

This approach is effective because it offers comprehensive care that addresses not only physical symptoms but also emotional, social, and spiritual needs. For instance, a patient with advanced cancer might receive pain management from a physician, counseling from a psychologist, spiritual support from a chaplain, and social assistance from a social worker, all coordinated to ensure that the patient and their family receive holistic care [8].

Dying with dignity, autonomy, and legacy

Saunders emphasized the importance of dying with dignity, believing that patients should have control over their end-of-life decisions, including where and how they wish to die. Hospice care, which Saunders pioneered, enables patients to spend their final days in a familiar, comforting environment, often at home, surrounded by loved ones [9]. This not only provides peace and comfort but also respects the patients' autonomy, allowing them to make informed decisions about their care [10].

Hospice care also plays a critical role in helping patients create a meaningful legacy and come to terms with their mortality. Providers can support patients in reflecting on their lives, accepting their situation, and leaving something meaningful behind for their families. This might involve creating memory boxes, writing letters, or simply spending time with loved ones. These practices ensure that patients are remembered for who they were, not just for how they died [11].

Integration of palliative care into other specialties

Palliative care has increasingly been integrated into various medical specialties, and its value in managing chronic and serious illnesses has been recognized. Oncology, in particular, has seen significant incorporation of palliative care principles, with many cancer centers now offering palliative care as a standard part of treatment [12]. Early involvement of palliative care in treating cancer patients has been shown to improve both quality of life and survival rates [13].

Other specialties, such as cardiology, pulmonology, and neurology, have also recognized the benefits of palliative care. For example, patients with advanced heart failure, chronic obstructive pulmonary disease (COPD), and neurodegenerative diseases like amyotrophic lateral sclerosis (ALS) are increasingly receiving palliative care alongside curative treatments [14]. This ensures that symptom management, psychosocial support, and advance care planning are integral parts of the overall treatment plan, improving patient outcomes and satisfaction [15].

Current impact and future directions

Palliative care is now an essential component of healthcare systems worldwide. The principles of palliative care have been integrated into the national healthcare policies of many countries, and there is a growing recognition of its importance in medical education and training [16]. The number of palliative care clinics and programs continues to increase, with over 1,700 in the United States alone [17]. These programs not only improve the quality of life for patients but also reduce healthcare costs by decreasing hospitalizations and the need for intensive treatments [18].

**Figure 1:**
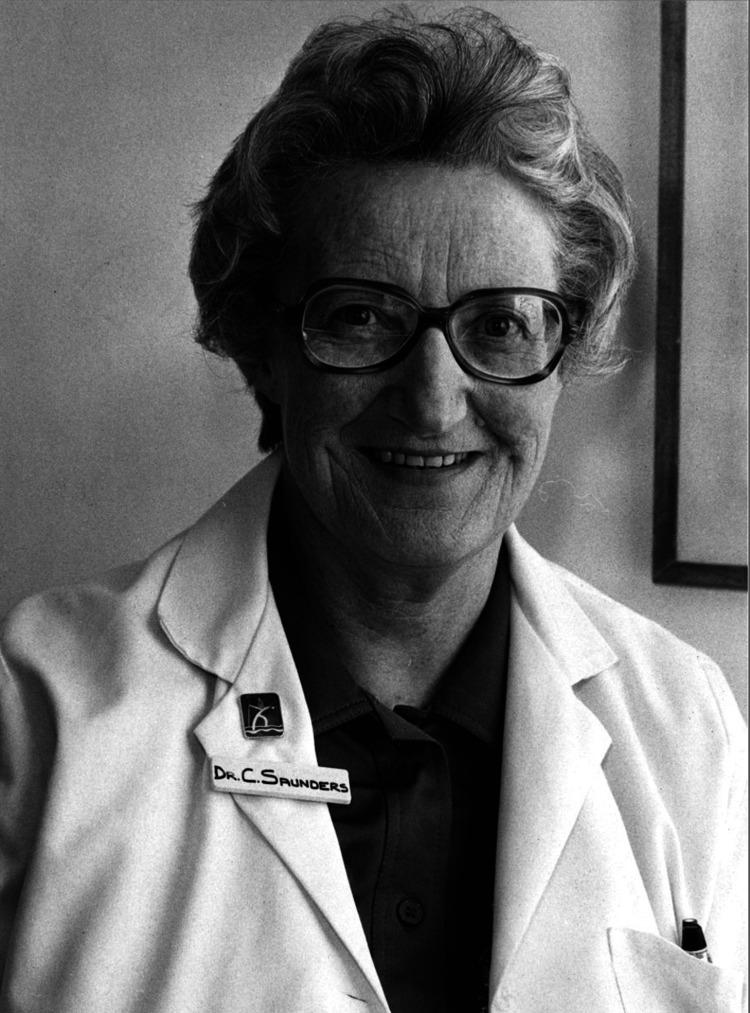
Dr. Cecily Saunders From the Cecily Saunders archive at https://cicelysaundersarchive.wordpress.com. Permission obtained from the publisher via Rightslink.

As the global population ages and chronic diseases become more prevalent, the demand for palliative care services will continue to rise. Future directions in palliative care will likely focus on expanding access to services, particularly in underserved areas, and integrating palliative care more fully into primary care and community settings [19]. Continued research is needed to refine pain management techniques, better understand the psychosocial aspects of terminal illness, and develop innovative care models adaptable to various healthcare systems [20]. Dr. Saunders' legacy is catalogued in the Cecily Saunders archive (Figure 1).

## Conclusions

Dame Cicely Saunders’ impact on medicine is profound. Her pioneering work in palliative care has transformed how healthcare professionals approach the treatment of patients with life-limiting illnesses. The principles she established have not only improved the quality of life for countless patients, but have also influenced the development of healthcare policies and practices globally. Saunders’ legacy endures in the thousands of palliative care programs and clinics that continue to provide compassionate and holistic care to patients and their families. Her work has ensured that palliative care is now a fundamental part of the global healthcare landscape, benefiting patients across medical specialties.
